# Supplementary material for: Deletion of wfs1 Impairs Oligodendrocyte Precursor Cells Dorsal Distribution and Myelination Through the wfs1-hmgcs1 Axis in Zebrafish
Source: Biology (Basel). 2026 Aug 16;15(16):1402. doi: 10.3390/biology15161402 (PMC13510151; doi:10.3390/biology15161402)
Supplement: Supplementary file 1 [file biology-15-01402-s001.zip › File S1-Transcriptome sequencing data and differentially expressed genes (DEGs) in wfs1-- zebrafish at 5 dpf/2.KEGG/wfs1KOvsWT/DOWN/src/dre04144.html]

dre04144


- dre:406837

- Down regulated genes

406837(-8.90108217929408)

- dre:387529

- Down regulated genes

387529(-1.06556993062178)

- dre:30671

- Down regulated genes

560210(-2.35591332225681)

- dre:550542

- Down regulated genes

550542\_1(-6.05661837629532)

- dre:336426

- Down regulated genes

336426\_1(-6.2697721375186)

- dre:793158

- Down regulated genes

793158(-6.94943122774273)

- dre:792828

- Down regulated genes

792828(-4.67911285540239)

- dre:492487

- Down regulated genes

492487(-1.20965091860504)

- dre:568170

- Down regulated genes

568170\_2(-3.04184048977547)

- dre:386856

- Down regulated genes

386856\_1(-1.99487147218035) novel.946(-6.7676180476835)
- dre:571431

- Down regulated genes

571431(-6.42862549702529)
- dre:245702

- Down regulated genes

245702\_1(-1.45433245048201)

- dre:792229

- Down regulated genes

792229(-6.97839628308154)

- dre:436718

- Down regulated genes

436718\_1(-6.85571323128554)

- dre:474327

- Down regulated genes

474327\_1(-5.01402477912525)

- dre:100003902

- Down regulated genes

100003902\_1(-2.55932971100136)

- dre:406684

- Down regulated genes

406684(-1.34842151914407)
- dre:393881

- Down regulated genes

393881\_1(-2.5844343493966)

- dre:100151129

- Down regulated genes

100151129(-4.46454752659211)

- dre:492487

- Down regulated genes

492487(-1.20965091860504)

- dre:100003902

- Down regulated genes

100003902\_1(-2.55932971100136)

- dre:445305

- Down regulated genes

445305\_1(-1.98867996431405)

- dre:445305

- Down regulated genes

445305\_1(-1.98867996431405)

Close
